# Knowledge translation in child welfare—improving educational outcomes for children at risk: study protocol for a hybrid randomized controlled pragmatic trial

**DOI:** 10.1186/s13063-018-3079-4

**Published:** 2018-12-29

**Authors:** Thomas Engell, Ingvild Barbara Follestad, Anne Andersen, Kristine Amlund Hagen

**Affiliations:** 1 The Regional Centre for Child and Adolescent Mental Health, Eastern and Southern Norway, PO Box 4623, 0405 Oslo, Norway; 2 The Norwegian Center for Child Behavioral Development (NCCBD), a University of Oslo affiliate, Postboks 7053 Majorstuen, 0306 Oslo, Norway

**Keywords:** Effectiveness study, Hybrid study, Knowledge translation, Implementation, Academic support, Child welfare, Education, Primary school children, Core components, Common elements

## Abstract

**Background:**

In Norway, a disproportionately high number of children receiving Child Welfare Services (CWS) struggle academically and drop out of school. Academic attainment is one of the strongest protective factors against societal marginalization. The present study is part of a knowledge translation project in collaboration with local CWS with the aim to develop, implement, and evaluate Enhanced Academic Support (EAS) for primary school children in CWS.

**Methods/design:**

The study is a mixed-methods hybrid type 2 randomized, controlled pragmatic trial. The participants are approximately 120 children whose families receive support measures from three child welfare agencies in and around Oslo, Norway, and practitioners from these agencies. Families are randomly assigned to either the EAS condition or “business as usual” support. Primary outcomes are math and reading skills, parental involvement in school, and intervention fidelity. Questionnaires and academic tests are administered at baseline, post-intervention (after 6 months), and at follow-up (after 12 months). Implementation drivers are assessed before and after the trial period, and intervention fidelity is monitored during the trial through checklists and structured telephone interviews. Semi-structured interviews and focus groups are conducted after the trial.

**Discussion:**

This hybrid study has two implications. (1) The effects of providing EAS to children in child welfare will be investigated. The study also explores how each core component of the intervention and the use of specific adaptations, implementation drivers, and other important child-level covariates moderate the overall effects. The results can provide valuable knowledge about how to deliver precise and effective academic support to increase academic skills and prevent dropout. In turn, this can promote academic completion and well-being, outcomes that are beneficial for both children and society at large. (2) The study also evaluates the feasibility of applying an Integrated Knowledge Translation model designed to develop, implement, and evaluate research-supported practice in health, care, and welfare services in less time than is usually the case. If deemed successful, this model will provide an efficient collaborative approach to translate the best available evidence into effective evidence-based practice, applicable in effectiveness research and quality improvement efforts.

**Trial registration:**

ISRCTN, ISRCTN38968073. Registered on 18 September 2017. 10.1186/ISRCTN38968073.

**Electronic supplementary material:**

The online version of this article (10.1186/s13063-018-3079-4) contains supplementary material, which is available to authorized users.

## Background

### Translating knowledge into effective and sustained practice

The translation of knowledge from research into effective and sustained practice is a critical issue in health, care, and welfare systems [1]. More successful translational efforts will likely result in improved services for patients, clients, and users and less inadequate treatment and care [2]. Municipal health, care, and welfare services in Norway experience increasing demands to ensure safe and effective services of high quality. Steps toward meeting these demands likely include identification of factors that contribute to favorable outcomes, supply and translation of the best available knowledge, and the establishment of quality monitoring and feedback systems.

### Need for knowledge translation in Child Welfare Services

In Norway, the Child Welfare Services (CWS) need support to succeed in quality improvement endeavors. The majority (approximately 70%) of CWS is delivered by municipal agencies located across the country [3]. These agencies vary considerably in size and organizational structure. They differ in terms of methods of practice, approaches for quality improvement, and quality monitoring systems. Although a state-led body governs and serves the municipal CWS agencies, the responsibility for ensuring and improving quality of services rests with local municipal government and the agencies themselves. CWS agencies often juggle demanding directives, high caseloads, scarce resources, and a high rate of staff turnover. Their practitioners hold challenging jobs and are prone to stress and burnout [4]. The Norwegian Child Welfare Act, section 4-4 [5] states: “The child welfare service shall contribute to provide the individual child with sound circumstances and opportunities for development by providing advice, guidance and assistance.” To meet increasing demands to ensure safe, effective, and high-quality advice, guidance, and assistance, municipal child welfare agencies would benefit from professional support.

### Marginalization and academic achievement among children in child welfare

Contributing to a healthy upbringing is important in itself, but it is also a good investment socially and economically. As a group, children in families who receive CWS are at greater risk of developing mental health issues and behavioral and substance abuse problems, and are also at greater risk for future unemployment and engaging in criminal behavior [6].

Children in child welfare in Norway are more than twice as likely to drop out of school compared to their peers [7]. Only two in ten children who have been involved with CWS complete secondary school on schedule, and 35% are neither employed nor in education by the time they reach 23 years of age [7]. In comparison, six in ten children in the general population complete secondary school on time, and under 10% are neither employed nor in education at the age of 24 years [8]. Children in CWS are often found to have knowledge gaps very early on in their academic careers, deficits that over time grow bigger and frequently result in academic failure and dropout [9]. Additionally, individual factors such as mental health, social skills, and executive functioning are likely to affect these children’s ability to succeed academically [10, 11]. Academic achievement is one of the strongest protective factors against later marginalization [12, 13]. In a study of 7000 Swedish children with a history of foster care, academic achievement strongly predicted positive outcomes in adulthood (i.e., not being on welfare, and showing less illness, drug abuse, and criminal behavior), even when other factors such as socioeconomic status were controlled [9]. Most studies on the provision of academic support to children in the CWS have focused on children in foster care [14–16]. Recently published statistics in Norway, however, show that children involved with CWS who are living with their biological parents are at a similar risk of academic failure as children who are placed outside the home [7].

Practitioners in CWS have reported that the children in their care need more appropriate and tailored support to succeed academically [17]. However, child welfare agencies lack the methods, training, and allocated resources to provide academic support. Research has indicated that providing academic support to children and their families outside of school hours, and especially at home, has very useful potential [14, 15]. Meta-analyses have shown that positive parental involvement (e.g., homework support, parent-teacher communication, positive communication about school, positive parental expectations) affects children’s academic performance positively [18, 19]. A systematic review of out-of-school-time academic (OSTA) programs for children at risk of dropout in the USA found that reading- and math-focused OSTA programs can improve reading and math achievement [20]. The authors highlighted the need to combine OSTA programs with other educational, community, and family support to achieve sustained effects.

### Using Integrated Knowledge Translation to develop and evaluate academic support in child welfare

To support child welfare agencies in the development of appropriate academic support, the current project applied an Integrated Knowledge Translation (IKT) model in collaboration with three child welfare agencies. IKT is an approach to research that engages researchers and stakeholders (e.g., child welfare managers and practitioners, youth and parents with child welfare experience, and school personnel) in collaborative partnerships to exchange, create, and utilize knowledge to address research issues [21]. The IKT model applied in the present project has combined IKT principles with methods from quality improvement and innovations in knowledge synthesis (we have labeled our model IKT-K, to distinguish it from other knowledge translation approaches). IKT-K entails synthesizing the best available evidence and translating the evidence into locally tailored and flexible research-based practice. IKT-K is structured in five phases: synthesis, co-creation, implementation, evaluation, and sustainment or de-implementation. During the first three phases, a locally tailored academic support intervention (Enhanced Academic Support, EAS) was developed based on common elements of effective academic interventions. EAS was implemented in three child welfare agencies, and its effects on academic achievement and parental involvement will be evaluated in this randomized controlled trial. The trial also evaluates the quality of EAS implementation and feasibility of the IKT-K model.

### Aims and hypotheses

The present study has three overarching aims:

1. To evaluate the feasibility of the IKT-K model designed to develop, implement, and evaluate empirically supported practice in CWS
2. To evaluate the effects of the intervention, EAS, on children in CWS and their families
3. To explore associations between implementation drivers (readiness, climate, fidelity) and outcomes for children and families.

The following research questions will be examined to evaluate Aim 1:

- To what degree are the core components of EAS implemented in the CWS?
- What adaptations are made to the core components of EAS?
- What are stakeholders’ perceptions of the IKT-K model’s feasibility and usefulness, as assessed in focus groups?
- To what degree are climate for implementing evidence-based practice (EBP) and susceptibility for change of practice (readiness for change) associated with intervention fidelity in the CWS?
- To what degree will practitioners in the experimental group increase their perceived competence in delivering academic support to children and families from pre- to post-intervention?
- To what degree is adherence to core components of EAS associated with academic achievement and parental involvement for families in the EAS group?

The following hypotheses will be tested to evaluate Aims 2 and 3:

- Children in families who receive the EAS intervention will improve their academic achievement relative to children and families in a parallel, active comparison group who receive “business as usual” (BAU) support.
- Parents who receive the EAS intervention will increase their engagement in their children’s school situation relative to parents who receive BAU.
- Intervention effects will be moderated by child age, readiness for change, and climate for implementing EBP.
- Covariates include children’s mental health, social skills, and executive functioning scores, as well as child gender and pre-intervention academic performance (math and reading) and parental involvement.
- Intervention effects (measured by academic performance tests and parental involvement) are associated with climate for implementing EBP and readiness for change.
- More adherence to EAS principles will be positively associated with academic achievement and parental involvement for families in the EAS group

## Methods and design

**Fig. 1:**
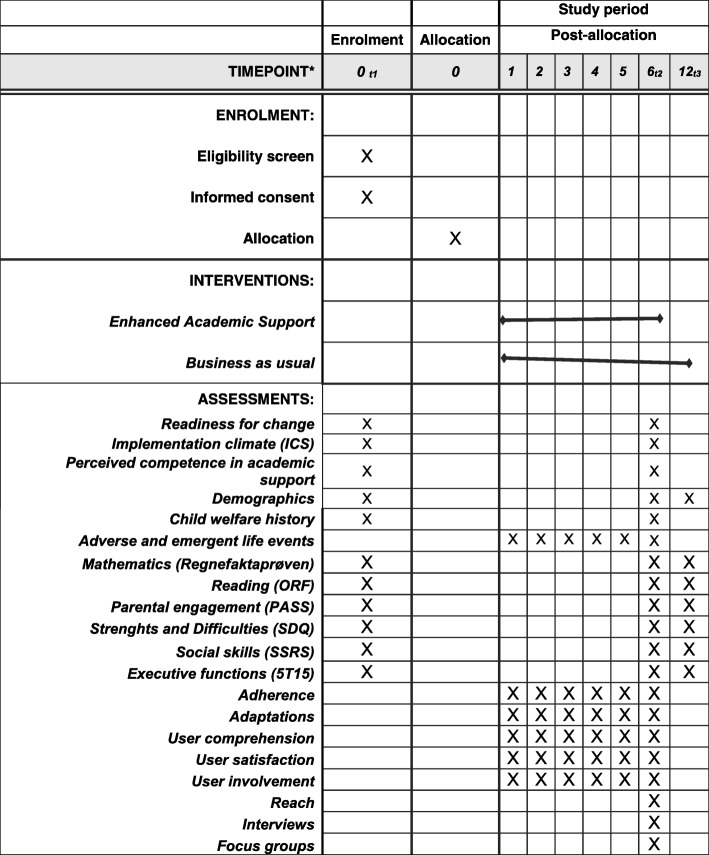
Schedule of enrollment, interventions, and assessments

This study is a randomized controlled pragmatic trial conducted in three ordinary child welfare agencies in and around Oslo, Norway. The agencies differ in size, organizational structure, and demographic characteristics. Selected practitioners at each site have received training in the EAS intervention. Practitioner selection to EAS training was mostly a matter of practicality (i.e., half of the practitioners in a team, geographic area, or unit were selected by their managers to receive training). Participating families are recruited individually at each site and randomized either to an EAS-trained practitioner or to a practitioner not trained in EAS who is delivering regular child welfare support measures (BAU). EAS is delivered over the course of 6 months. Participants are assessed before and immediately after EAS, and at follow-up, 6 months after the end of the intervention. The schedule of recruitment, allocation, assessments, and experimental conditions is provided in Fig. 1. The Standard Protocol Items: Recommendations for Interventional Trials (SPIRIT) checklist is provided as Additional file 1.

### Participants

Eligible participants are boys and girls in primary school and their parents, whose family receives support measures from CWS. We plan to recruit 120 children and their parents.

#### Inclusion criteria

The following inclusion criteria must be met:

1. Children in families receiving support measures from municipal CWS
2. Boys and girls in the 1st to 7th grades and their parents/caregivers
3. Parents/caregivers who give informed consent. Consent, assent, and the questionnaires are available in Norwegian, English, Arabic, and Somali.

#### Exclusion criteria

The exclusion criteria are as follows:

1. Developmental disability
2. Parents/caregivers not able to give informed consent due to language restriction (not able to understand Norwegian, English, Arabic, or Somali)
3. One child only from each family can participate

The study also includes managers and practitioners at the local child welfare agencies (*N* = 160), the children’s teachers, and other stakeholders (parents and youths who previously received CWS, youth from user organizations, and local school counselors; estimated *N* = 22) in and around Oslo, Norway.

#### Power calculation

The study aims to recruit 120 families. The intervention under study is newly developed; hence, we used a meta-analysis testing the overall effect of similar interventions (targeting parental involvement in children’s learning, including “paired reading”) to inform the required sample size. The outcomes used to assess effect sizes in the meta-analysis were a combination of different standardized and unstandardized reading measures. The suggested effect sizes were in the range of *d* = .65 to 1.15 [22]. With α < 0.5, power = 0.80, and expected effect size *d* = .65, the necessary sample size is *n* = 78, with 39 families in each group. Although the Oral Reading Fluency (ORF) test administered in the current trial is similar to the assessments used by the studies in the meta-analysis, none of those studies used the actual ORF test of this trial. Hence, the power calculation lacks some precision. To account for uncertainty in the power calculation, subgroup analyses (of gender, site, and CWS measure), and possible study dropouts, and to compensate for the possibility of non-normal distribution of scores, more participants than deemed necessary according to the power calculation were recruited. Based on information from the participating CWS agencies about their target groups, this sample size seems attainable.

### Knowledge translation procedure

The first phase of the IKT-K model, the synthesis phase, started in January 2016.

In the *synthesis phase*, an adapted common elements methodology [23, 24] was applied to identify common practice, process, and implementation elements (*N* = 166 elements) of interventions with a significant positive effect on academic attainment for children at risk of school dropout. A systematic review was conducted [25]. All information available about the effective interventions (*N* = 31) was reviewed by coders and plotted as elements in a matrix created to compare frequencies. Frequency-based algorithms were applied to identify common elements of effective interventions and common combinations of these. The four most common elements were selected as core components and used in the development of the academic support intervention. The frequency with which these core components appeared in non-effective interventions or interventions with negative effects was also reviewed, and their given weight of importance was adjusted accordingly. Algorithms were also applied to extract process and implementation elements most frequently used in combination with common elements. Taken together, the results of these analyses pointed to specific practice elements (e.g., actions or activities), their rate of involvement in effective interventions, methods of effective delivery, recipient characteristics, delivery conditions, and promising combinations of elements (a manuscript on the methodology and results is in preparation).

In the *co-creation phase* and through a series of workshops, facilitated teams consisting of researchers, an education specialist, a coordinator, CWS practitioners, users (parents and youths), school personnel, and other stakeholders collaborated in developing a locally tailored academic support intervention (EAS) based on the common elements profiles. The teams also prepared the training program and local implementation plans, and made pragmatic adjustments to the research design.

Prior to tailoring the implementation plan, an assessment of the climate for implementing evidence-based practice and readiness to change was conducted in each CWS. Each phase of the IKT-K model includes specific implementation strategies designed to overcome typical barriers to implementation and sustainment. Assessments of climate and readiness were used to identify particularly prominent or unpredicted barriers and facilitators which warranted increased effort or additional strategies.

In the *implementation phase*, the implementation was prepared, the training program was conducted, recruiters and assessors were trained, and the intervention and research infrastructures were piloted. Particularly engaged practitioners and managers were offered roles as site champions and given additional training in the EAS intervention and knowledge translation. Champions were assigned roles and responsibilities such as coordination, ongoing coaching, following up of recruiters, leadership engagement, etc. Adaptations were made based on feedback from practitioners and other stakeholders during piloting.

In the *evaluation phase*, a hybrid type 2 pragmatic trial will be applied to evaluate the effectiveness of EAS and the feasibility of the IKT-K model. The term “hybrid type 2” refers to research designs that evaluate clinical (or behavioral or educational) interventions and implementation strategies simultaneously [26]. Focus groups and semi-structured interviews with practitioners, users, and other stakeholders will be conducted to gain further understanding of the feasibility and usefulness of both EAS and the IKT-K model.

In the *sustainment or de-implementation phase*, data from the evaluation phase will inform an overall evaluation of EAS together with the co-creation teams. In collaboration, these teams will decide whether to carry out sustainment and improvement strategies or de-implementation strategies.

### Intervention

Practitioners who deliver the EAS intervention have participated in a 14-h training program in EAS. The training consisted of approximately 50% didactic education, 20% role play, and 30% discussions, problem solving, and dialogue. Trained practitioners tried out the delivery of EAS during the 5 months of piloting. They have also participated in a full day booster session, and a second booster session is planned after 6 months of recruitment to the study. The practitioners receive ongoing coaching from local EAS champions at each site and from the external implementation team running the study (The Regional Centre for Child and Adolescent Mental Health, Eastern and Southern Norway, study authors KAH, AA, IBF, TE) upon request. The amount of coaching is monitored. The practitioners have received an EAS handbook, in addition to various pedagogical, educational, and planning materials to be used with children and families.

EAS consists of four core components: (1) guidance in positive parental involvement in school, (2) structured tutoring in reading and math, (3) guidance in homework structure and routines, and (4) guidance in positive reinforcement, praise, and feedback. These four components are delivered to families in six sessions over a period of 6 months with support and follow-up between sessions. The sessions are delivered at home visits, or in other settings at the family’s preference. The first session is assigned to build rapport with the family and to identify goals together with the family, and one session at the end is assigned to evaluate, repeat material as needed, and create a sustainment plan with the family. The four sessions in between are assigned to each core component.

Each of the visits consists of specific actions and activities (practice elements) for the practitioners and the families to engage in together. Practitioners are also instructed on how these actions and activities ought to be carried out (process elements). Important implementation elements such as ongoing support, local tailoring, and intervention flexibility are integrated into the delivery of the intervention. Even though core components are assigned to separate sessions, and practice and process elements are clearly described, flexibility within fidelity is encouraged. This means that practitioners can adapt the sequence of components, emphasis on components, combinations of components, and time between sessions as they see fit, as long as they adhere to the practice and process elements of the core components and report adherence, dosage, and adaptations in the monitoring checklists after each session (see the section “Monitoring and safety”). Additionally, pre-defined component-specific adaptations that are likely to be useful in different settings and scenarios are described and encouraged in training and in the handbook.

EAS is designed to be a flexible supplement to the support that families in CWS already receive. Hence, EAS is delivered in addition to the family’s child welfare measure. The practitioners are free to combine EAS sessions with other measures or help they provide, or they can deliver EAS in separate sessions with the family. A session usually varies in length from 30 to 120 min. The session length is monitored.

### Comparison condition (business as usual)

The comparison condition is “business as usual” (BAU) in Norwegian CWS. The content, structure, and length of BAU vary among agencies and among individual practitioners. Children and families in the BAU condition have been assigned a practitioner who has not received EAS training but who follows the family and offers regular support measures. These measures may include advice and guidance, parent training, financial aid, parent relief, etc. Meetings can take place both at the families’ homes and other settings, such as the child welfare office or the school. BAU can also include some academic support, typically in the form of facilitating parent-teacher communication or the use of homework support at the school or in the community. Information about services provided in the BAU condition is collected using end-of-intervention-checklists (see the subsection “Implementation measures”).

### Implementation strategies

The following tables describe implementation strategies that are either planned, in progress, and/or completed in the study using current guidelines for reporting implementation studies [27, 28]. Table 1 describes implementation actors, and Table 2 describes implementation strategies. To categorize which level each strategy targets, categorization based on a dynamic adaptation process (DAP) framework [29] is used (involving system, organization, provider, and client levels). The first seven strategies are integral in the IKT-K model. Additional strategies are applied based on the intervention, context assessments, and knowledge exchanged between stakeholders in co-creation teams.

**Table 1:** Actors involved in implementation strategies

Delivery system actors	• Site champions^a^ • Site staff and practitioners
Support system actors	• External implementation team^b^ • Co-creation teams, one team for each of the three CWS sites^c^
Synthesis and translation system actors	• External implementation team • Co-creation team

^a^Managers, practitioners

^b^Researchers, educator, coordinator, research assistants

^c^Practitioners, managers, user representatives (youths and parents), researcher, educator, coordinator, facilitator

**Table 2:** Description of implementation strategies

Strategy	Classification	Category of actor(s)	Action	Dose	Action target (determinant and level)	Temporality^a^	Outcome measure
*Integral strategies in the Integrated Knowledge Translation ( IKT-K) model*							
Engage stakeholders and utilize local knowledge	Process strategy	All systems	Stakeholders are in collaborative partnership to address mutually understood need for practice improvement		To utilize local knowledge on all levels and facilitate stakeholder buy-in and ownership on provider and organization levels	All	Feasibility
Use facilitation	Process strategy	Support system	An assigned facilitator objectively guides co-creation discussions, promotes knowledge exchange, and minds equal participation and power imbalances	Five 4-h workshops^b^ with each co-creation team, additional meetings if necessary	Facilitate collaborative problem-solving and promote mutual consultations among stakeholders to ensure integration of different forms of knowledge on organizational and provider levels	Co-creation, implementation, evaluation, and sustainment	Feasibility
Develop glossary	Process and dissemination strategy	Support system	Develop a glossary of frequent, difficult, and potentially ambiguous terms, and ban potentially offensive terms. Align project documents with glossary	4-h introduction workshop with each co-creation team	Promote equal understanding and participation and prevent the use of offensive terms. At organizational level	Co-creation	Feasibility
Assess context	Process strategy	Delivery system and support system	Implementation climate and readiness for change assessed by online survey to all staff. Determinants discussed in co-creation teams	10–15-min online survey, 4-h implementation workshop with each co-creation team	To assess readiness and identify barriers and facilitators to implementation at all levels	Co-creation	Climate for implementing evidence-based practice (EBP), readiness for change
Tailor intervention	Process strategy	Support system and delivery system	Local knowledge and experience utilized to tailor components of the intervention to fit daily practice and address needs at each site	Two 4-h intervention workshops with each co-creation team, feedback from practitioners during and after training and piloting. One 4-h adjustments workshop	To develop a feasible and appropriate intervention on client, provider, and organizational levels	Co-creation	Feasibility, appropriateness, acceptability, fidelity, reach
Tailor strategies	Process strategy	Support system	Local knowledge and experience utilized to tailor implementation strategies to context	4-h implementation workshop with each co-creation team. 4-h adjustments workshop	To tailor strategies to address barriers and leverage facilitators identified through context assessments and knowledge exchange in co-creation teams. On provider and organizational levels	Co-creation	Feasibility, fidelity, reach
Develop a formal implementation plan	Integration and capacity-building strategy	Delivery- and support system	A formal implementation plan has been created describing implementation infrastructure, goals, procedures, strategies, and adaptations to each site	One formal implementation plan, site-specific implementation manuals with adaptations to each site, continuous registration of adaptations	Guide and organize implementation processes on organizational and provider levels with appropriate and structured adaptations	Developed during co-creation, adaptations throughout all phases	Feasibility, fidelity, reach
*Additional strategies based on the intervention, context assessment, and knowledge exchange in co-creation teams*							
Make intervention dynamic and flexible	Dissemination strategy	Synthesis and translation system	Common elements of effective interventions have been used as basis for the intervention components to enable dynamic and flexible delivery	Systematic review, common elements analysis	To utilize the best available empirical evidence and improve intervention feasibility, appropriateness, and acceptability at all levels	Synthesis, co-creation	Feasibility, appropriateness, acceptability, fidelity
Train champions	Capacity-building strategy	Support system and delivery system	Champions have received additional training in the intervention, knowledge translation, implementation strategies, and behavior change	7-h group training	Build local implementation and coaching capacity on provider and organizational levels	Implementation	Feasibility, fidelity, reach
Use ongoing coaching	Capacity-building strategy and integration strategy	Delivery system and support system	Intervention practitioners receive group coaching from external implementation team and champions. Individual coaching is provided upon request or in cases of fidelity drift	Bimonthly from external implementation team, monthly from champions, and individually on request (registered)	Promote learning and integration of the intervention in practitioners and champions on provider and organizational levels	Implementation, evaluation, sustainment	Fidelity, reach, perceived competence
Use continuous support	Capacity-building and integration strategy	Support system	Group consultations, booster sessions, telephone support, training of new practitioners, recruiters, and champions	Bimonthly meetings with practitioners, recruiters, and champions, 4-h booster session every 6 months, telephone support, visiting support and training on request (registered)	Provide support and boost engagement, implementation quality^c^, and recruitment at the provider and organizational levels	Implementation, evaluation, sustainment	Feasibility, fidelity, reach, perceived competence
Develop contingency plans	Capacity-building strategy	Support system and delivery	In cases of turnover, sick leaves, fidelity drift, or adverse events, specific plans of engagement are described for champions in their implementation plans	Use monitored	Prepare and plan for barriers and other events that threaten implementation at organizational and provider levels	Developed during co-creation, adaptations throughout all phases	Feasibility, fidelity, reach
Develop and distribute educational material	Integration strategy	Support system	Created and distributed intervention handbooks, planning material, and pedagogical material	Approx. 50 handbooks, 150 copies of planning material, 200 copies of sponsored reading and math material	Promote intervention implementation and effectiveness at the provider and client levels	Developed in co-creation, used in implementation, evaluation, and sustainment	Acceptability and appropriateness of material, fidelity, primary effectiveness outcomes
Develop and distribute implementation resources	Capacity-building strategy	Support system	Created and distributed implementation and recruitment manuals, implementation checklists/posters to champions, and recruitment flyers in 4 languages	Three site-specific implementation manuals, recruitment manuals, and implementation checklists/posters, approx. 500 flyers	Promote recruitment at client level and EAS implementation quality^c^ at organizational and provider levels	Developed in co-creation, used in implementation and evaluation	Feasibility, fidelity, reach
Use implementation audit and feedback	Integration strategy	Support system	Double-informant measures of fidelity, user satisfaction and user involvement	Audit after each intervention session. Group-level feedback to practitioners bimonthly	Motivate and engage practitioners and prevent fidelity drift at the provider level	Evaluation	Fidelity, primary effectiveness outcomes

^a^Phases of IKT-K: synthesis, co-creation, implementation, evaluation, sustainment

^b^Total co-creation workshops (all 4 h); 3 introduction workshops, 6 intervention workshops, 3 implementation workshops, 3 adjustments workshops

^c^Implementation quality should be understood as the degree to which Enhanced Academic Support (EAS) reaches the target population, is used with adherence, competence, and appropriate adaptations by practitioners, and is comprehended by parents and children

### Measures

The primary implementation measures are related to intervention fidelity (adherence to core components, parent comprehension of core components, and user satisfaction with delivery of intervention components). Primary effectiveness outcomes are reading and math scores and parental involvement in school. The ORF test has two subscales: a fluency score and an accuracy score. A composite variable of the two reading outcomes will be made.

**Fig. 2:**
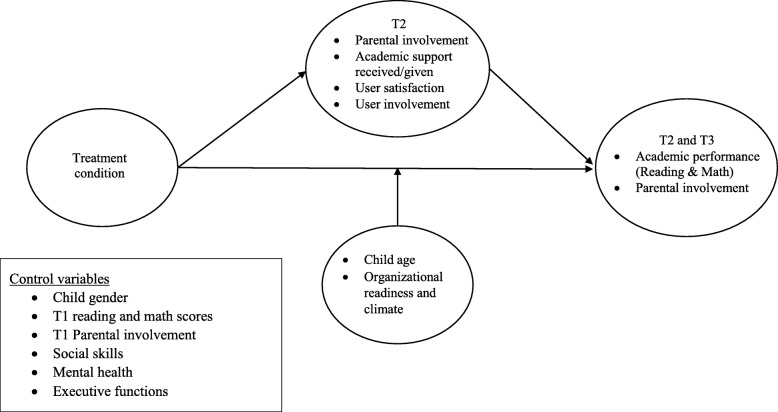
Model of intervention effects, covariates, mediators, and moderators

Secondary outcomes (and covariates) are measures of intervention feasibility, acceptability, and appropriateness; practitioners’ perceived competence in providing academic support; and children’s mental health and adjustment, social skills, and executive functioning. The theoretical implementation model is shown in Fig. 2.

**Fig. 3:**
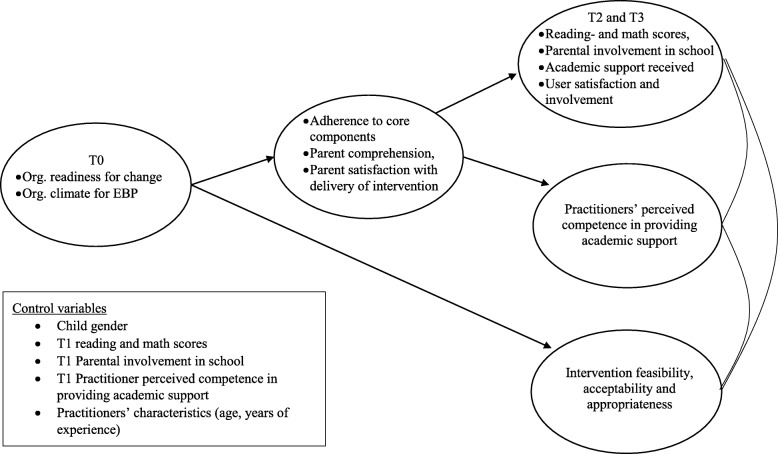
Model of implementation

Organizational readiness for change and organizational climate for implementing EBP are measured to inform the implementation process and to be tested as predictors in the implementation model (see Fig. 3).

#### Implementation measures

The following implementation measures are used in this study:

- A monitoring checklist has been developed to measure adherence to core components, dosage, competence in delivery, parent comprehension of core components, adaptations, and user involvement and satisfaction with delivery of intervention components. The checklist is completed by EAS practitioners using smartphones after each intervention session and by parents answering the same questions in telephone interviews after each intervention session (see the section “Monitoring and safety” for more details).
- An end-of-intervention checklist measures academic support received/given, emergent life events/adverse events, and overall user satisfaction and involvement during the last 6 months (intervention period). It has been developed specifically for this study and is administered at the post-assessment to parents (15 items), children (16 items), and practitioners (20 items) in both conditions. Items are rated on a 4-point scale (“not at all”, “to a small degree”, “to some degree”, “to a large degree”). Each version ends with an open question about any additional information to be answered in free text.
- Intervention feasibility, acceptability, and appropriateness will be measured using three four-item scales: Acceptability of Intervention Measure (AIM), Intervention Appropriateness Measure (IAM), and Feasibility of Intervention Measure (FIM) [30]. The AIM, IAM, and FIM are completed by the EAS practitioners post-intervention.
- Organizational readiness for change is assessed using an unpublished questionnaire made by the study authors with 32 items rated on a 5-point scale (ranging from “strongly disagree”, “somewhat disagree”, “unsure”, “somewhat agree”, to “strongly agree”). The questionnaire measures organizational factors, characteristics, needs, and work climate as well as staff characteristics, qualities, and needs. It is administered to all employees at all CWS sites, pre- and post-intervention. Organizational managers answer two additional items measuring organizational resources and opportunities.
- Organizational climate for implementing evidence-based practice (EBP) is assessed using the Implementation Climate Scale (ICS) [31]. The scale consists of 18 items rated on a 5-point scale (from “not at all” to “very great extent”). The ICS assesses the degree to which there is a strategic organizational climate supportive of EBP implementation. Subscales include focus on EBP, educational support for EBP, recognition for EBP, rewards for EBP, selection (employment) for EBP, and selection (employment) for openness. The ICS is administered to all employees at all CWS sites pre- and post-intervention. It has been validated with practitioners in 32 mental health organizations and 12 child welfare services in the USA [31, 32].
- Practitioners’ perceived competence in providing academic support is measured using an unpublished questionnaire developed by the study authors. It includes 12 items rated on a 5-point scale ranging from “strongly disagree” (0) to strongly “agree” (5). The questionnaire assesses knowledge and use of competencies relevant to the core components of EAS without using the specific wording of the core components in the EAS handbook. It is administered pre-training and post- intervention to EAS practitioners.
- CWS employees’ perceptions of the implementation process and the EAS intervention will be gauged by conducting focus group interviews post-intervention. An interview protocol will be prepared based on the IKT-K model and the DAP framework, including questions about the appropriateness and acceptability of EAS. Some of the topics to be discussed in the focus groups will include results from the quantitative data analyses.
- Semi-structured interviews will be conducted with a selection of participating children and parents after the intervention to learn more about their experiences with EAS. To select families to approach for participation in interviews, a randomization procedure in the Confirmit software will be used. Randomization will be stratified to select families who benefited from the intervention, who did not benefit from the intervention, and families with different ethnicities from each of the three sites.

#### Effectiveness measures

The effectiveness measures used in the study are described as follows:

- The *Oral Reading Fluency (ORF)* test [33] measures children’s reading abilities. The test consists of three short passages that are grade level- and season-sensitive (i.e., there are different passages for fall, winter, and spring). Children are asked to read the text aloud to the interviewer. Each reading sequence is timed to one minute. The interviewer monitors the reading and alerts the child when the time is up. The number of errors and the number of words read are recorded. The median scores of both errors and number of words read from the three passages are used. The test gives a score for fluency and a score for accuracy in reading. It is normed for children in the 2nd to 5th grades in Norway. The ORF test is administered to children at the pre-intervention, post-intervention, and follow-up assessments.
- The *Test of Arithmetic* (*“Regnefaktaprøven”*), developed by the University of Stavanger, Norway, is used to measure the children’s mathematical abilities. It consists of two sets of addition problems and two sets of subtraction problems (each set with a different difficulty level) and one set each of multiplication and division problems. Children are asked to complete as many problems within a 2-min timeframe as they can. The number of correct answers is tallied up. The Test of Arithmetic is normed for Norwegian children in each grade level of elementary school and is administered to children at the pre-intervention, post-intervention, and follow-up assessments.
- Parental involvement in school is assessed using the *Parent and School Survey (PASS)* [34], a 24-item survey scored on a 5-point Likert scale administered to parents. The PASS questionnaire asks parents to indicate how involved they are in their children’s schoolwork, school activities, and collaboration with school personnel. It is administered at the pre-intervention, post-intervention, and follow-up assessments.

#### Secondary measures and covariates

The following are the secondary measures and covariates used in the study:

- The *Strengths and Difficulties Questionnaire (SDQ)* [35–37] is a 25-item questionnaire that measures emotional problems, behavioral problems, hyperactivity, difficulties with peers, and prosocial behavior. Each item is rated on a 3-point scale (0 = “not true”, 1 = “sometimes true”, 2 = “certainly true”). The SDQ has a child/youth, parent, and teacher version. It also consists of an impact score that measures the degree of negative influence any problems have on different aspects of the child’s daily life (such as family activities and learning at school). Large population studies using the SDQ have been conducted in Norway [38, 39]. Regional norms for children and youth in Norway are available. The SDQ will be administered to children, parents, and teachers at the pre-intervention, post-intervention, and follow-up assessments.
- The *Social Skills Rating System (SSRS)* [40] is a standardized, multi-rater instrument that assesses social skills in children. It is administered to children, parents, and teachers. The children’s version has 34 items divided into four subscales: cooperation, assertion, empathy, and self-control. The parent scale includes 38 items measuring cooperation, self-esteem, responsibility, and self-control. The teacher’s version has 30 items assessing cooperation, self-esteem, and self-control. Each item is rated on a 4-point Likert scale, ranging from 1 (“never”) to 4 (“very often”). The SSRS has been used with Norwegian samples in earlier studies [41], and the teacher’s version has been validated and normed for children and adolescents in Norway [42]. The SSRS will be administered to children, parents, and teachers at the pre-intervention, post-intervention, and follow-up assessments
- *Five-to-Fifteen* [43] is a 181-item questionnaire developed to assess attention deficit hyperactivity disorder (ADHD), common comorbid conditions to ADHD, and associated problems in children and youth aged 5 to 17 years. The present study uses four subscales of the questionnaire with a total of 28 items which assess executive functions (attention and concentration, overactivity and impulsivity, passivity/inactivity, and planning/organizing). Items are rated on a 3-point scale (“does not apply”, “applies sometimes/to some extent”, “applies”) and are administered to parents and teachers at the pre-intervention, post-intervention, and follow-up assessments. The parent version of Five-to-Fifteen has been validated and normed with Nordic samples (Swedish, Danish, and Finnish) with acceptable psychometric properties [44]. The teacher version has been validated and normed in Danish samples with acceptable psychometric properties [45].
- *Demographics and background information.* Parents answer questions about their age, gender, marital status, pregnancy, ethnicity, education, occupation, living arrangements, income, relocation during the last 5 years, other children in the household, and whether they receive help from any health, care, or welfare service. Parents also answer questions about the child’s gender, age, and school grade, and if the child receives help from any other health, care, or welfare service. The child answers questions about his or her age and gender. Demographic information is collected at the pre-intervention, post-intervention, and follow-up-assessments. Background information about the family’s history of child welfare service (current and previous child welfare measures) is obtained from child welfare practitioners with parental consent.

### Procedures

#### Referral

Children and their families are referred to a child welfare agency by notification of concern (e.g., by teachers, community nurses, physicians, police, or others). The agency either opens a case of inspection or dismisses the note of concern. If probable concern is established, but not in terms of out-of-home placement recommendation, the family is offered support measures from CWS. If the family accepts, they are eligible for study inclusion if they fulfill the inclusion criteria.

#### Enrollment

At that point, a case worker at the child welfare agency reviews the family’s eligibility. If they are eligible, the case worker provides neutral information about the study and asks if the family is interested in participating. If they are interested, the case worker asks for oral consent to provide the research staff with the family’s contact information.

If consent is given, the research coordinator calls the parent and provides more information about the study and answers questions.

#### Consent

A home visit is scheduled to complete the recruitment and pre-assessments. A trained interviewer visits the family and provides detailed information about the study to both parents (if they are both present) and child. The interviewer reviews eligibility, verbal assent is collected from the child, and written, informed consent is collected from parents electronically on iPads. The parent is also asked to give consent to allow the child welfare practitioners to receive an oral summary of assessment results and for the research team to contact the child’s teacher. Consents and questionnaires are available in Norwegian, English, Arabic, and Somali.

#### Pre-assessment

Directly after consent, pre-assessments commence. The parent and child are each handed an iPad to answer questionnaires, and the interviewer administers the reading and math assessments on paper with the child. The pre-assessments take about 60 min to complete. After completion, an email with a link is sent to the child’s primary teacher providing information about the study, an invitation to answer questionnaires, and the secure online questionnaires. Within a week after pre-assessments, an oral summary of results from the assessments of reading and math skills, mental health, social skills, and executive functions is provided to the family’s assigned child welfare practitioner with the parent’s permission. The post-intervention and follow-up assessments are also conducted in home visits by an interviewer.

#### Randomization

At the time of consent, parents and children are informed that they will be randomly allocated to one of two conditions; one group, the BAU condition, receives regular measures from CWS, whereas the experimental condition receives the EAS intervention in addition to a regular child welfare measure. Blinding is not possible in this study; child welfare practitioners who have received EAS training will exclusively give EAS to study families, and parents and children will most likely understand to which group they have been assigned.

After completing the pre-assessment, participants are automatically randomized to either the intervention group (EAS) or the comparison group (BAU). A computer software (Confirmit) generates a random numbers table to assign random numbers to participants within blocks. A block randomization with a block of 10 is used, and randomization is carried out site-wise. The research coordinator informs the team manager at the site to assign the case to a practitioner with or without EAS training. All edit trails in Confirmit are recorded. Outcome assessors are blinded to allocation. In-depth technical details can be provided upon request.

#### Intervention: Enhanced Academic Support (EAS)

Families allocated to the intervention group are assigned a practitioner with training in EAS. EAS is delivered as described in the “Methods and design” subsection “Intervention”.

#### Comparison condition: business as usual (BAU)

Families allocated to the BAU group are assigned a practitioner without training in EAS. BAU is delivered as described in the “Methods and design” subsection “Comparison condition (business as usual)”.

#### Post-assessment

Six months after pre-assessment, the post-assessment is administered. A selection of participating families will be invited to semi-structured interviews, and a selection of participating practitioners and other stakeholders will be invited to participate in focus group interviews.

#### Follow-up assessment

Six months after post-assessment, the follow-up assessment is administered.

### Statistical analyses

We will consider efficacy for each of the primary outcomes. In other words, efficacy will be gauged in an outcomes-specific manner. A significance level of .05 will be used.

Outcomes will be evaluated using analysis of covariance (ANCOVA), controlling for baseline scores and covariates. Children’s age, implementation drivers, children’s mental health, social skills, and executive functions will be tested in regression models, as will possible subgroup analyses. To test for indirect effects (or mediation), models will be tested in a structural equation modeling (SEM) framework. Indirect effects variables include PASS, Five-to-Fifteen , and the end-of-intervention checklist measures. Implementation drivers as predictors of outcomes will be tested in regression models. We will evaluate effectiveness in two parts. The pre-post outcome analysis will use ANCOVA with baseline measures and covariates as control variables. We will test intervention effects including all data waves in SEM. See Figs. 2 and 3 for the theory of intervention change depicting variables included in the analyses.

We will examine and present data both in intention-to-treat (ITT) and as-treated (AT) designs, as the two approaches answer different questions. An ITT design answers the question “Does the intervention make a difference?” An AT analysis, on the other hand, answers the question “What are the effects likely to be if the client (or family) is exposed to the intervention?” We consider both of these questions important. This procedure has been recommended as best practice [46].

We will use multiple imputation for missing values for the pre-post ANCOVA in an ITT design. In the SEM models, missing data will be estimated using full information maximum likelihood.

## Monitoring and safety

### Audit and feedback

After each session of EAS with the family, the practitioner completes a dynamic fidelity checklist on their smartphone/tablet or computer using an online survey. The survey takes about 5–10 min to complete, depending on the number of core components and adaptations that were used in the session. After completion, an automated reminder is sent to the project coordinator, and an available interviewer calls the parent and conducts a structured telephone interview. Two additional attempts are made if the parent does not answer the call. The interviewer uses an online survey to retrieve an interview guide and plot the parent’s answers. The interview guide is based on the checklist the practitioner recently completed. That is, detailed questions are only asked about the core components that the practitioner stated were used in the last session. Additionally, parents are asked if they remember doing something else in the session. If they mention another core component, they are asked detailed questions about that as well. The interview is structured this way to limit the amount of questions asked to practitioners and parents and to prevent attrition due to long checklists and interviews. The total number of questions available to practitioners is 113; however, an average checklist requires 25–30 answers (minimum 19). An average parent interview contains 20–25 questions (minimum 14).

#### Variables audited

The variables audited are duration of session, contact since last session, adherence to core components, parents’ and children’s comprehension of core components, use of pre-defined adaptations of core components, use of additional adaptations to core components (free text), client satisfaction, client involvement in decisions, and adverse events or relevant emergent life events.

#### Feedback

Monitoring data from each site are aggregated bimonthly and used as feedback to the practitioners. KAH and TE, together with the site champions, deliver feedback on team meetings. On request from the practitioners, they can receive individual feedback to use in ongoing coaching with the site champions. Emphasis is placed on adherence to core components, frequently used adaptations, and client satisfaction. Group-level feedback reports are also delivered via newsletters.

In cases of severe drift, serious adverse events, or repeatedly poor client satisfaction, KAH and TE will confer with site champions to commence one of the following contingency plans: additional booster training with the team, individual booster training with a practitioner, or gathering of the local EAS practitioners to discuss additional adaptation or change.

### Stopping rules (discontinuation criteria)

The following criteria are considered grounds for discontinuation:

- If a family’s regular child welfare measure is concluded or terminated, and the family is no longer receiving child welfare support
- If the CWS practitioners or data collectors uncover acute suicidality, psychosis, abuse, or other conditions that render the EAS intervention and data collection not viable, safe, or ethical
- If the CWS practitioners uncover any serious adverse effects of the EAS intervention, rendering it unsafe to offer clients
- If the child is placed out of home and/or parents lose custody of the child
- Withdrawn consent from the study
- Withdrawn government funding (the Research Council of Norway)
- Breach of ethical standards or regulations.

In cases of dropout or discontinuation, the family will be asked to complete post-assessment if it is deemed ethical and viewed as appropriate by the family’s child welfare case worker.

### Data management

The Regional Centre for Child and Adolescent Mental Health, Eastern and Southern Norway has a license and data management agreement with Confirmit. All electronic data are collected using the web-based tool Confirmit Authoring, and all websites used to collect data are encrypted with a security clearance. Data on paper are stored in a secure safe with access restricted to authorized research personnel. All data are stored in accordance with standards and regulations set by the ethics committee Norwegian Centre for Research Data (NSD). Sensitive information is stored separately from directly identifiable information. An identification key is stored electronically in a secure database. Only authorized research personnel have access to the key. Further information can be provided upon request.

### Handling and follow-up of adverse events, data monitoring committee (DMC)

This study is an effectiveness trial conducted in existing child welfare agencies, not in a research facility. The intervention is considered low risk and no more intrusive than what is normally being delivered to children and families in CWS. The participating agencies have internal procedures for detecting, reporting, and following up on any adverse events in their clients. The study does not pose any restrictions on the agencies’ internal procedures for handling adverse events or offering other services if deemed appropriate. In the event of the agency terminating a family’s EAS intervention, the research team will be informed, and post-assessment will be conducted if deemed appropriate by the agency. In cases of perceived risk of adverse harm inflicted on people or property, research personnel will report to authority, abiding by law. The trial is not blinded, as the practitioners know what kind of service they offer the children and their families. No interim analyses will be conducted, in order not to bias the progression of the study. For these reasons, the current study has not appointed a DMC.

### Access to source data

The Regional Centre for Child and Adolescent Mental Health ensures that the investigator/institution will permit trial-related monitoring, audits, reviews, and regulatory inspections, by providing direct access to source data/documents if needed, and that such inspections do not violate the rights and/or anonymity of trial participants, including children, their families, their therapists, or other CWS employees.

## Discussion

This hybrid study has two main aims: (1) to evaluate the feasibility of an Integrated Knowledge Translation (IKT-K) model used in Child Welfare Services (CWS) designed to develop, implement, and evaluate empirically supported practice; and (2) to test the effectiveness of Enhanced Academic Support (EAS), a home-based intervention to improve academic achievement in children and their families in child welfare.

Advances in implementation science have outlined strategies that are likely to be pivotal to succeed in translational efforts, such as developing collaborative partnerships with stakeholders, using facilitation, and adapting and tailoring to context [47]. Similar strategies have long been used in the field of quality improvement, such as co-creation (or co-production) methods [48], often combined with iterative process models designed for continuous improvement [49]. The IKT-K model applied in this study attempts to utilize the best available evidence from implementation science together with established quality improvement methods to advance knowledge translational efforts. Involving stakeholders in mutually dependent partnerships is an integral strategy in this study, operationalized using facilitated co-creation approaches to locally tailor adaptable aspects of the study. The aim of these strategies is to utilize local knowledge and expertise, ensure buy-in from stakeholders, and thus promote acceptability, implementation, and sustainment of the newly introduced practice change. If feasible, this model can offer a pragmatic, efficient, and usable approach to development, implementation, and sustainment of evidence-based practice, which in turn can support knowledge translation and quality improvement in health, care, and welfare services.

Providing effective academic support to children in CWS can be of great value to both individual families and society at large. The need for academic support in child welfare populations is extensive, and CWS agencies are required to contribute to helping these children academically. However, these agencies are not provided with additional resources to deliver academic support to the families they serve. Hence, they need means to deliver academic support that fits within their current practice. EAS is a pragmatic intervention tailored to child welfare daily practice by the CWS agencies using it. Building the intervention around core components offers a much-needed flexibility that enables child welfare practitioner to incorporate empirically supported academic support within their existing practice. EAS requires limited training and resources and, if effective, could prove a highly cost-effective intervention given the large returns successful investments in education can provide for individuals and society.

### Trial status

Recruitment commenced in January 2018, and the trial is currently in progress. The estimated completion date of the trial is December 2019.

## Additional file

Additional file 1: SPIRIT 2013 checklist: recommended items to address in a clinical trial protocol and related documents. (DOC 121 kb)

## Abbreviations

BAU: Business as usual
CWS: Child Welfare Services
EAS: Enhanced Academic Support
EBP: Evidence-based practice
ICS: Implementation Climate Scale
IKT: Integrated Knowledge Translation
IKT-K: Integrated Knowledge Translation-KOBA model
KOBA: Kunnskapsoverføring i Barnevernet
PASS: Parent and School Survey
SDQ: Strengths and Difficulties Questionnaire
SSRS: Social Skills Rating System
